# Looking for Plant microRNAs in Human Blood Samples: Bioinformatics Evidence and Perspectives

**DOI:** 10.1007/s11130-023-01063-9

**Published:** 2023-05-31

**Authors:** Lorenzo Olmi, Gerardo Pepe, Manuela Helmer-Citterich, Antonella Canini, Angelo Gismondi

**Affiliations:** Dept. Biology, University of Rome Tor Vergata, Via della Ricerca Scientifica 1, Rome, 00133 Italy

**Keywords:** Plant food, Diet, Gene therapy, Cross-kingdom regulation, Plasma

## Abstract

Literature has proposed the existence of a cross kingdom regulation (CRK) between human and plants. In this context, microRNAs present in edible plants would be acquired through diet by the consumer’s organism and transported via bloodstream to tissues, where they would modulate gene expression. However, the validity of this phenomenon is strongly debated; indeed, some scholars have discussed both the methodologies and the results obtained in previous works. To date, only one study has performed a bioinformatics analysis on small RNA-sequencing data for checking the presence of plant miRNAs (pmiRNAs) in human plasma. For that investigation, the lack of reliable controls, which led to the misidentification of human RNAs as pmiRNAs, has been deeply criticized. Thus, in the present contribution, we aim to demonstrate the existence of pmiRNAs in human blood, adopting a bioinformatics approach characterized by more stringent conditions and filtering. The information obtained from 380 experiments produced in 5 different next generation sequencing (NGS) projects was examined, revealing the presence of 350 circulating pmiRNAs across the analysed data set. Although one of the NGS projects shows results likely to be attributed to sample contamination, the others appear to provide reliable support for the acquisition of pmiRNAs through diet. To infer the potential biological activity of the identified pmiRNAs, we predicted their putative human mRNA targets, finding with great surprise that they appear to be mainly involved in neurogenesis and nervous system development. Unfortunately, no *consensus* was identified within the sequences of detected pmiRNAs, in order to justify their stability or capability to be preserved in human plasma. We believe that the issue regarding CKR still needs further clarifications, even if the present findings would offer a solid support that this hypothesis is not impossible.

**Supplementary Information:**

The online version contains supplementary material available at 10.1007/s11130-023-01063-9.

## Introduction

MicroRNAs (miRNAs) are short (21–24 nucleotides), single-stranded, non-coding RNA molecules involved in the post-transcriptional regulation of gene expression.

MiRNAs are produced endogenously by a wide variety of organisms, ranging from viruses to mammals and plants [1]. They are synthetized in the nucleus as a primary transcript, called pri-miRNA, which undergoes several processing steps, including import into the cytoplasm, to become a mature miRNA [2]. In this form, it can be loaded on a dedicated RNA-induced silencing complex (RISC), which binds a messenger RNA (via base complementarity between transcript sequence and stacked miRNA) and promotes its degradation or translational inhibition [3]. In plants, miRNAs would seem more prone to induce the instability of the target rather than its translation arrest [1]. Despite the mechanism primarily attributed to miRNAs being the negative regulation of their targets, literature has documented that they may also act as enhancers of translation under specific circumstances [4, 5]. Plant miRNAs (pmiRNAs) are very resistant to extracellular conditions, including low pH (e.g. gastric environment), temperature variation (e.g. boiling), enzyme activity (e.g. ribonucleases), due to several factors such as: (i) the association with shielding components (e.g., Argonaute proteins, sugars, secondary metabolites); (ii) the inclusion into plasma membrane-derived vesicles (i.e., exosomes); (iii) the peculiar content/disposition in GC; (iv) the methylation in position 2′-OH of the sugar residue at the 3′-terminal nucleotide (which confers protection against exonuclease degradation and 3′-uridylation) [6–14]. All this evidence would sustain the hypothesis that exogenous miRNAs may survive for long time and be transferred from one organism to another, giving rise to a phenomenon known as cross-kingdom regulation (CKR). The existence of a pmiRNAs-mediated CKR of the animal gene expression has been recorded by several scholars [15–19], and particular interest has been devoted to clarifying whether these small nucleic acids may be effectively absorbed in the gastro-intestinal tract of mammals and then reach consumers’ tissues via the bloodstream. To date, several studies have reported the presence of pmiRNAs in samples of mammal tissue, serum, and plasma [20–24]. Despite these findings, other researchers have failed to replicate the results obtained by Liang and colleagues [25, 26]; thus, some authors have suggested that the presence of putative pmiRNAs in human plasma may be explained as artifacts or contamination [27], rather than absorption by diet. Even if pmiRNAs were effectively absorbed and transported in the bloodstream, another critique, moved by Cottrill and Chan [28] and also reported in an exchange of opinion between Witwer & Zhang [29], is that the amount of these molecules probably wouldn’t be enough to carry out a detectable regulatory effect on the consumer’s gene expression. To date, only one study has been performed with the aim to detect the presence of pmiRNAs in small-RNA Next Generation Sequencing (NGS) data of human plasma [30]. However, the results of this study have been contested by Witwer [31], who showed that the putative pmiRNAs found by Liu and colleagues [30] had perfect matches with portions of the human ribosomal RNA, suggesting that those sequences should more likely be considered as by-products of human RNA degradation than diet-derived miRNAs. Another similar study has been performed on sequencing data from mammalian (human and porcine) breast-milk exosomes [32]. Here, the authors have applied more stringent conditions and filtering, identifying several pmiRNAs. However, it is clear that the body of evidence should be expanded to strongly corroborate CKR theory.

Identifying plant-derived miRNAs in human sequencing data without ambiguity is, therefore, a hard task, which requires special attention to avoid false positives. However, the achievement of this goal would be very interesting for providing clear evidence about the stability of pmiRNAs in human blood and would represent a milestone in support of the existence of a diet dependent CKR.

According to all these premises, in this work we aim to build a reliable and reproducible bioinformatics protocol for identifying putative pmiRNAs in small-RNA sequencing data from human plasma. In order to avoid any misinterpretation and considering literature recommendations, several checks were applied to our bioinformatics process, such as filtering of NGS data on human genome, transcriptome, and extra-chromosomal DNA and matching of putative pmiRNAs with miRBase and human RefSeq RNA databases to exclude false positives.

## Materials and Methods

Materials and methods applied in this work are reported in detail in Supplemental Material S0.

## Results and Discussion

The fact that over 30% of human coding genes is estimated to be a target for endogenous miRNAs denotes the importance of these molecules in the regulation of gene expression [33]. Thus, showing that also pmiRNAs introduced by diet may take part in this phenomenon would be an amazing finding, able to open new perspectives on the relationship between food and health.

To date, the effectiveness of synthetic and/or natural pmiRNAs in modulating animal gene expression has been suggested and documented by several scholars [15–19, 34]. However, concerns about pmiRNA transfer, stability, and activity in animal cells and organisms have been raised [25, 26]. Therefore, if the acquisition of dietary pmiRNAs in human blood was confirmed and correctly validated, a key evidence supporting the existence of CKR and proposing miRNA delivery via oral administration during gene therapies would be provided.

For this reason, in the present contribution, our aim is to obtain solid results about the capability of pmiRNAs to be preserved and transported in human plasma, using smallRNA-sequencing data from previously published research. Indeed, to date, the literature focused on the same objectives [30] has been imputed with lack of reliability, due to the absence of significant controls [31]. Thus, every step of the current analysis was performed in order to ensure the maximum possible stringency and avoid mistakes in pmiRNAs identification. Our approach cannot of course distinguish whether the presence of exogenous miRNAs in the human plasma is ascribable to absorption by diet or contamination by operators but, being able to consistently identify plant-derived sequences in most of the samples we processed, the obtained data would certainly provide an interesting step further in this debate.

**Fig. 1:**
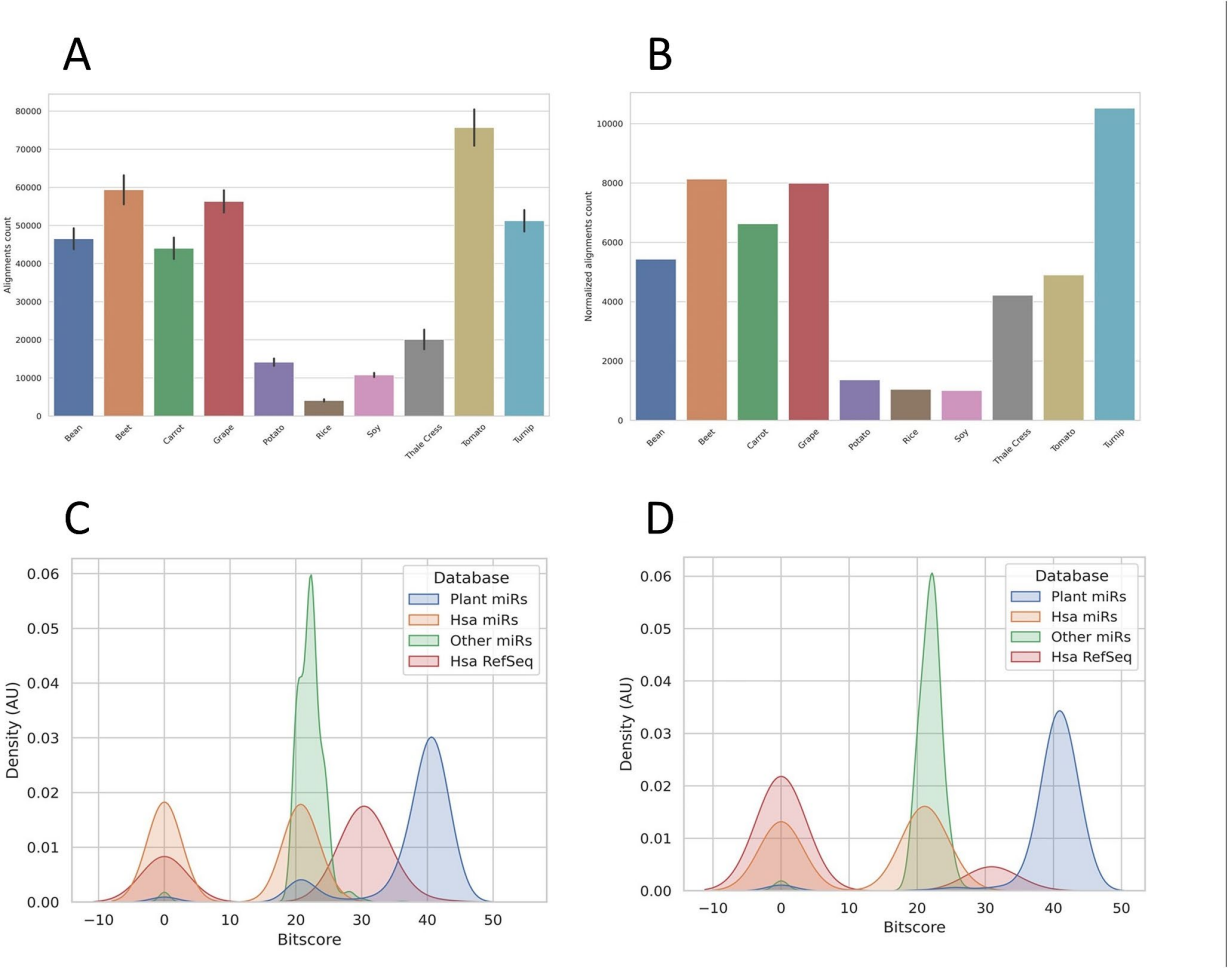
A) Bar plot showing the average amount of reads (± standard deviation) which successfully aligned to different plant genomes (*Beta vulgaris* L. – Beet; *Brassica rapa* L. *–* Turnip; *Daucus carota* L. *–* Carrot; *Glycine max* (L.) Merrill *–* Soy; *Oryza sativa* L. – Rice; *Phaseolus vulgaris* L. – Bean; *Solanum lycopersicum* L. – Tomato; *Solanum tuberosum* L. – Potato; *Vitis vinifera* L. – Grape; *Arabidopsis thaliana* (L.) Heynh - Thale cress). B) Average amount of reads which aligned to plant genomes, after normalization for the size of the different plant genomes. C) Kernel Density Estimate (KDE) for BitScores distributions obtained searching for similarity of putative pmiRNAs against different databases using BLAST was shown (AU: arbitrary units). D) KDE with the same information of panel C but after filtering out ambiguous reads showing 100% identity with human sequences or the lowest BitScore in association with known pmiRNAs.

First, reads were filtered on human references, as stated in materials and methods section. After that, they were aligned to the genomes of 10 plant species, selected among those available, well-annotated, and presenting a role in human diet, plus *Arabidopsis thaliana* (L.) Heynh (the best-known plant model system). The average number of reads which aligned to each genome is shown in Fig. 1A. To check for any bias given by the different sizes of the genomes, the average alignment count of each plant species was normalized for the relative genome size. As reported in Fig. 1B, this parameter did not particularly affect the results, except for *Solanum lycopersicum* L. and *Beta vulgaris* L. This suggests that the alignments were not produced by mere chance, since an increase in the number of alignments proportional to the genome sizes was not observed.

After selecting reads that aligned to regions annotated as miRNA or pre-miRNA in the plant genomes, the filtered sequences were checked for similarity using BLAST+ against the following databases: (I) known pmiRNAs from miRBase; (II) known human miRNAs from miRBase; (III) known miRNAs of the remaining species from miRBase; (IV) human RefSeq mRNA. The alignment score for each sequence with the accessions registered in the 4 databases was expressed as BitScore value and their distribution reported in the kernel density estimate (KDE) of Fig. 1C. At this point, reads which showed 100% identity with human sequences or did not have their highest BitScore in association with known pmiRNAs were discarded. Indeed, before filtering out ambiguous results, a big superimposition of known pmiRNAs distribution (blue) and Human mRNA RefSeq (red) could be observed. After this filtering, KDE resulted as reported in Fig. 1D, showing that the sorting process effectively diminished the chances for misidentification of pmiRNAs.

In total, the pipeline was able to identify 350 validated pmiRNAs, with an average amount ranging from 1.33 Reads per Million (RPM) to 0.005 RPM across all sequencing experiments (average 0.199 RPM; median 0.08 RPM). A detailed report, including sequences, RPM values, best matches with plant miRNomes, and other information on the verified pmiRNAs, can be viewed in Supplemental Material Table S1.

Beyond a peculiar structural difference (a methyl group in position 2′-OH of the sugar residue to the 3′-terminal nucleotides of pmiRNAs), animal and plant miRNAs diverge for target recognition. Indeed, the first ones bind their targets mainly at the 3′-UTR, while the second group presents complementarity sites located everywhere on the transcripts [34]. Taking into consideration these aspects, authenticated pmiRNAs were fed to psRNA-Target for predicting all their potential human mRNA targets (Supplemental Material Table S2). The obtained data were then passed to g:Profiler, with the aim to identify the pathways potentially influenced by the selected pmiRNAs. The fifteen most represented GO:BP (Gene Ontology: Biological Process) terms are shown in Table 1, with their own confidence score. Full g:Profiler output is available in Supplemental Material Table S3. The results showed that almost all mammalian molecular pathways putatively regulated by the pmiRNAs detected in plasma (GO:BP terms) were those involved in neurogenesis and nervous system development. This result was quite unexpected. Indeed, assuming CKR by dietary-acquired pmiRNAs plays a role in explaining the bioactivity of plant foods, one would have expected that the modulated pathways hinted signalling systems usually linked to nutraceutics (e.g., antioxidants). For example, pathways implicated in inflammatory processes, redox homeostasis, dysmetabolism, and even cancer would have been good points in favour of CKR hypothesis. However, our evidence could appear as extremely interesting: literature has mainly linked the beneficial effects of plants to their content in secondary metabolites (e.g., flavonoids), highlighting for the latter specific functions toward the above-mentioned biological processes [35, 36; see Supplemental Material S0], but it is possible that pmiRNAs introduced by diet exert other molecular functions still not identified and specifically linked to plant consumption. Our data do not invalidate CKR, but certainly open new questions about the possible role of this phenomenon in modulating human physiology and metabolism. Consequently, how can we explain our evidence? Is it possible that only pmiRNAs acting in specific human tissues and/or on certain targets are stored in blood? Can we hypothesize that, once ingested, pmiRNAs are rapidly absorbed by cells and only some of them remain in the vascular system? In this regard, another aspect to be considered is that pmiRNAs preserved in human sera belong to a subclass, maybe more stable, of all those absorbed by diet. The stability of these nucleic acids has been linked to several factors, such as binding to Argonaute proteins (AGOs), inclusion into exosomes, GC content/disposition, coupling with plant molecules (e.g., sugars, secondary metabolites) [6, 13]; therefore, to evaluate the issue, we tried to find a *consensus* sequence, a conserved core, and/or peculiar features for the pmiRNAs detected in our samples but, unfortunately, this goal failed. Nevertheless, it would be needed to investigate this aspect more in detail, to better clarify which agents are involved in pmiRNAs protection within human body.

**Table 1:** The 15 most likely molecular pathways influenced by the pmiRNAs detected in human plasma were identified by g:Profiler and here reported (the complete output was present in Supplemental Material Table S3). They were listed as GO:BP (Gene Ontology: Biological Process) terms, together with their confidence scores (indicated as -log_10_ of adjusted *p*-values)

Enriched GO:BP Term	-log_10_ of Adjusted *p*-value
Axon development	13.958
Synapse organization	13.431
Axonogenesis	12.618
Cell junction assembly	11.530
Modulation of chemical synaptic transmission	11.194
Regulation of trans-synaptic signaling	11.091
Regulation of synapse structure or activity	10.896
Regulation of nervous system development	10.536
Dendrite development	9.945
Regulation of synapse organization	9.886
Renal system development	9.761
Regulation of neuron projection development	9.739
Urogenital system development	9.344
Kidney development	9.239
Positive regulation of cellular component biogenesis	9.220

**Fig. 2:**
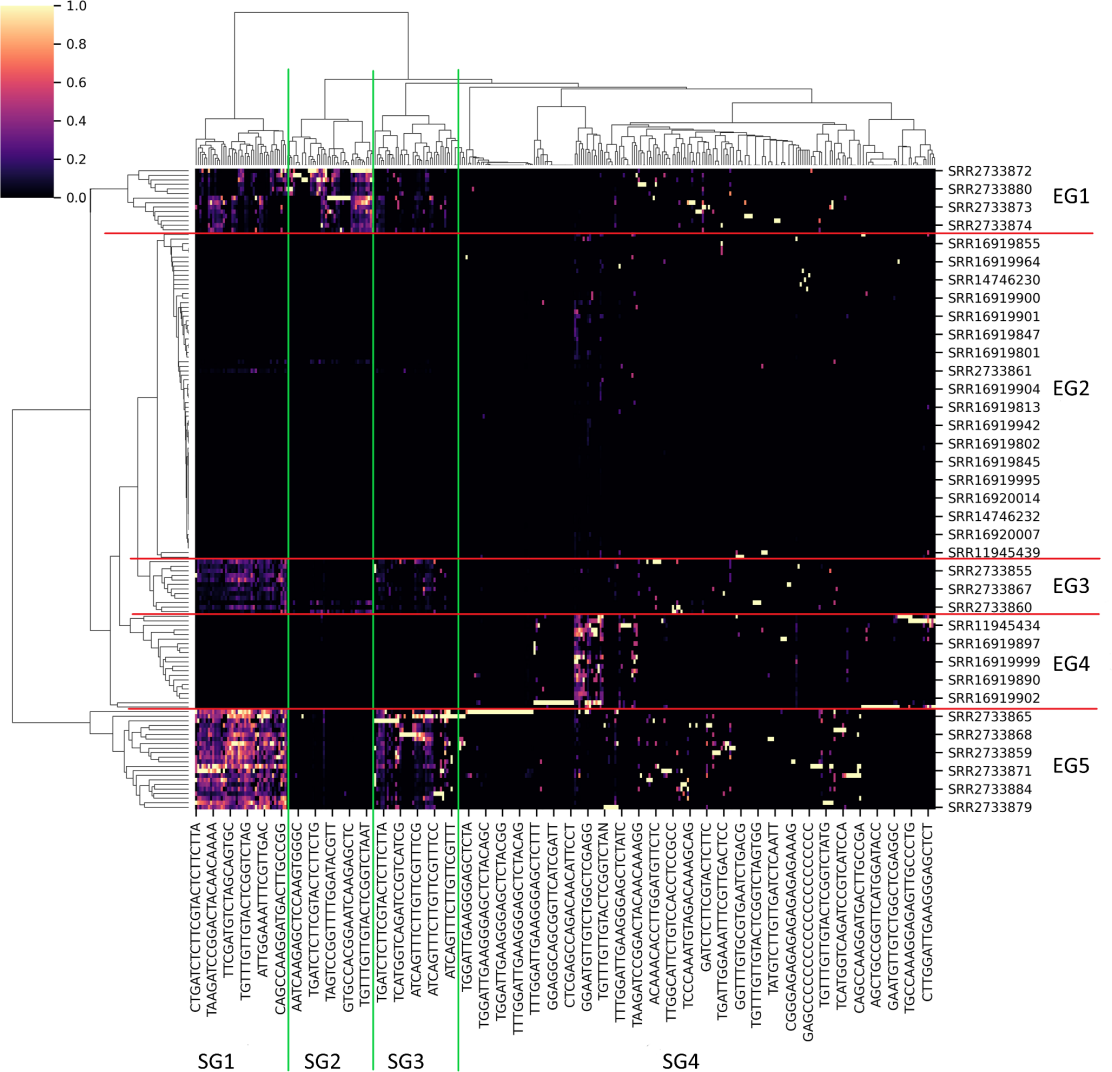
Heatmap and dendrograms representing the cluster analysis performed on the matrix of counts. From these results, 5 groups of experiments (EG1-5, delimited with red lines) and 4 groups of sequences (SG1-4, delimited with green lines) were identified and used for further evaluations. Only the names of some representative experiments and the sequences of some representative reads were reported in the figure, due to the high amount of them included in the analysis. The color scale is in arbitrary units and indicates the abundance of each pmiRNAs in the several experiments

Cluster analysis, based on the presence/absence and counting of pmiRNAs, showed that some plant sequences and individuals from sequencing experiments formed well-defined groups. Heatmap and dendrograms representing such type of information can be found in Fig. 2, where the detected groups were highlighted. We were able to identify 5 groups regarding the experiments (EG1-5, red delimitations) and 4 groups regarding the miRNA sequences (SG1-4, green delimitations). As evident from the heatmap, all EGs presented a well-defined pattern of pmiRNAs presence, with the only exception of EG2, where no specific pmiRNA profile was found and the abundance of each sequence appeared also very low. The clustering for EG1, 3, and 5 seemed mainly driven by the same sets of sequences (those included in SG1, 2, and 3). Surprisingly, we checked that EG1, 3, and 5 were totally constituted by experiments coming from the same projects, respectively. By contrast, EG2 and EG4 collected sequencing experiments from different projects. To better show this finding, the distribution of the data from each project was reported in Table 2 for all experimental groups (EG). Lacking any meaningful information about the individuals subjected to the analyses (e.g., diet; ethnicity; origin), two hypotheses can be proposed to explain the previous evidence: (i) contamination events occurred during sample preparation or sequencing for the projects whose data are included in EG1, 3, and 5; (ii) similar dietary habits for the individuals involved in those experiments determined a shared pmiRNA profile in their plasma. The possibility of grouping due to different miRNA isolation/sequencing approaches is unlikely to be considered. Of particular interest is the fact that sequencing data from all projects could be found only in EG2 and that EG4 included the results of 2 distinct projects (Table 2), suggesting that maybe these two groupings are surely free of any technical bias. Regarding the SGs, SG4 did not show a peculiar fingerprint, while the sequences collected in SG1, 2, and 3 were quite conserved in specific experimental sets (that is EG1, EG3, and EG5). Thus, for these miRNAs, the presence of a *consensus* was investigated, in order to associate their relative conservation rate in the individuals to specific sequences potentially able to provide higher stability, capability of inclusion in extracellular vesicles, and/or promotion of gastro-intestinal absorption. Anyway, no preserved region/core was revealed, discouraging our hypothesis. Another interesting aspect to bear in mind during the interpretation of the present results has been described by Liang and colleagues [21]. Here, the scholars have administered melon juice to patients and have observed a peak of pmiRNAs presence in subjects’ plasma after three hours from ingestion, which then gradually decreased. Given the fact that fasting is usually required in patients before taking blood samples, the lack of pmiRNAs in most experiments (i.e., those in EG2 and the ones excluded from clustering analysis) could be explained by this medical procedure.

**Table 2:** For each cluster of experiments (EG1-5), the origin of the data from the 5 small RNA sequencing projects (PRJNAs) used in this research was reported as percentage distribution (%)

	PRJNA637898	PRJNA779718	PRJNA735638	PRJNA299307	PRJNA593788
EG1	0	0	0	100.00	0
EG2	5.55	80.58	8.33	2.77	2.77
EG3	0	0	0	100.00	0
EG4	28.57	71.43	0	0	0
EG5	0	0	0	100.00	0

Another important issue that should be discussed is related to the final amount of pmiRNAs which can be detected in blood. Indeed, although some pmiRNAs were found in a relatively high number (i.e., mean of 1.33 RPM), most of sequences was present in a very small level, supporting the criticism that diet does not represent an effective way for delivering these molecules into hosts. In this regard, some authors have questioned the ability of low doses of food-derived miRNAs to significantly modulate consumers’ gene expression [28, 29]. Taking this observation into consideration, an eventual oral administration of pmiRNAs for experimental and/or therapeutic purposes should be characterized by high doses or a more efficient delivery and carrier system, such as synthetic exosomes. However, to be honest, it is important to report that literature has also documented that the activity of miRNAs depends on the concentration of the relative targets and not on their own abundance, being continuously recycled within the cytoplasm [34, 37–39; Supplemental Material S0]. Thus, this last evidence would indicate that all pmiRNAs detected in the current study from plasma samples might exert a potential biological role on their hosts.

## Conclusions

Our study provides evidence that pmiRNAs may be identified in human plasma by analysing small RNA sequencing data, although not in a consistent manner and generally in a very low amount. The high stringency of the method we implemented in the present research should clear out any doubt of false positives, thus offering reliable information about this issue. Regarding the origin of the identified pmiRNAs, we cannot demonstrate neither their acquisition by diet nor that their presence was caused by contamination, as this depends on sampling and sequencing protocols independent from us. However, the fact that most of the experiments displayed sequences identifiable as pmiRNAs hints at the eventuality that the absorption through diet of these nucleic acids is feasible. Unfortunately, we could not identify any *consensus* in the sequences of pmiRNAs found in the samples, suggesting that no sequence motif seems to be involved in the horizontal transfer between organisms. Surprisingly, the main putative human targets of the pmiRNAs detected in NGS dataset were factors associated to neurogenesis and nervous system development, opening novel and interesting hypothesis about the function, selection, and stability of pmiRNAs in human blood. We can conclude that the outcome reported in this contribution would seem to support the existence of CKR and the development of potential gene therapies based on food-derived pmiRNAs.

## Electronic Supplementary Material

Below is the link to the electronic supplementary material.

Supplementary Material 0

Supplementary Material 1

Supplementary Material 2

Supplementary Material 3

## Data Availability

The procedure reported in this research can be viewed in detail and reproduced by downloading and running the pmiRFinder tool from GitHub (https://github.com/lorenzo-bioinfo/pmirfinder). A detailed description on how to use the tool can be found in the documentation. The raw data analysed in this paper can be obtained from the papers mentioned in the Materials and Methods section, while the results were all included in the main text or in the relative supplemental material files.
