# Supplementary material for: Looking for Plant microRNAs in Human Blood Samples: Bioinformatics Evidence and Perspectives
Source: Plant Foods Hum Nutr. 2023 May 31;78(2):399–406. doi: 10.1007/s11130-023-01063-9 (PMC10363053; doi:10.1007/s11130-023-01063-9)
Supplement: Supplementary file 1 — Supplementary Material 0 [file 11130_2023_1063_MOESM0_ESM.docx]

**SUPPLEMENTAL MATERIAL S0**

**A) *MATERIALS AND METHODS* SECTION**

***Download and Quality Control of sequencing data***

Raw sequencing data for human plasma small RNA were downloaded from NCBI-SRA [1], using the SRA-Toolkit suite (https://trace.ncbi.nlm.nih.gov/Traces/sra/sra.cgi?view=software and the SRA Toolkit Development Team). A total of 380 sequencing experiments from 5 different projects (i.e., PRJNA299307 [2]; PRJNA637898 [3]; PRJNA779718 [4]; PRJNA593788 [5]; PRJNA735638 (unpublished)) were considered. Quality control of the *FastQ* files was performed using FastQC [6]. Reads were then trimmed using Trim Galore (https://github.com/FelixKrueger/TrimGalore) (keeping only reads ranging from 18 to 26 nucleotides in length) and checked again for quality.

***Removal of human sequences***

To exclude human sequences, reads were filtered on human known miRNAs, human genome, human extra-chromosomal regions, and human cDNA using Bowtie [7]. Reference data was downloaded from miRBase (https://www.mirbase.org/) and Ensembl FTP website (release 107; http://ftp.ensembl.org/pub/release-107/). To provide maximum stringency, no mismatch was allowed in the first 20 positions (full sequence when sequence length is less than 20) and reads with multiple alignments were discarded. Reads that failed to align to all the above references were temporarily considered non-human and, therefore, maintained for subsequent steps of analysis.

***Identification of putative pmiRNAs***

The genomes of 10 plant species, selected among those available, well-annotated, and presenting a role in human diet, were downloaded from Ensembl Plants (release 54; http://ftp.ensemblgenomes.org/pub/plants/release-54/). Those were: *Beta vulgaris* L. (Beet)*, Brassica rapa* L. (Turnip)*, Daucus carota* L. (Carrot)*, Glycine max* (L.) Merrill (Soy)*, Oryza sativa* L. (Rice)*, Phaseolus vulgaris* L. (Bean)*, Solanum lycopersicum* L. (Tomato)*, Solanum tuberosum* L. (Potato)*, Vitis vinifera* L. (Grape), *Arabidopsis thaliana* (L.) Heynh (Thale cress). Previously filtered reads were aligned to the selected plant genomes using Bowtie, with the same settings used for human references. Alignments were stored in SAM files. A Python script was employed (see Data Availability Statement) to identify only the reads aligning in regions annotated as *miRNA* or *pre-miRNA*. These reads were temporarily considered putative pmiRNAs and subjected to next analysis.

***pmiRNAs validation***

To reduce the possibility of misidentification, putative pmiRNAs were aligned to miRBase accessions and human RefSeq mRNAs using BLAST+ [8]. For each of them, the best matches against plant miRNAs, human miRNAs, other organisms’ miRNAs, and human RefSeq mRNAs were extracted and analysed. Bitscore is the measure used to evaluate the alignments quality, as it is independent of the database size. Putative pmiRNAs which showed a perfect match to portions of human mRNAs were discarded. Afterwards, sequences with the highest Bitscores (being those matching with known pmiRNAs) were kept, while the others were discarded. Sequences passing this filtering stage were considered putative pmiRNAs and their average amount across all sequencing experiments was reported as Reads per Million (RPM).

***Cluster analysis***

In order to perform a cluster analysis on the verified pmiRNAs for each sequencing experiment, two different matrices were generated: I) a *presence-absence* matrix, in which the presence of each pmiRNA in a specific sequencing experiment was encoded as 1 while its absence as 0; II) a *counts* matrix, where the normalized count of each verified pmiRNA was reported for each individual present in the experiment. In this second analysis, counts were normalized using the *MinMaxScaler()* function from the *sklearn.preprocessing* module [9]. By this way, the highest count for each experiment was set to 1, the lowest to 0, and the others scaled accordingly. The two matrices were then used to perform a clustering with a Python script, by *hierarchy.linkage()* from *scipy.cluster* module [10]. Using this function, Ward algorithm for variance minimization was used as linkage criterion [11] and distance measure was set on Euclidean. Data from these matrices was extracted and further processed as follows: groups of experiments were tested for over-representation of sequencing projects in each group; groups of sequences were tested for the presence of a *consensus* sequence in every single group. Sequencing experiments showing no pmiRNA were excluded from cluster analysis.

***Target prediction and functional analysis***

The list of verified pmiRNAs was used as input for psRNATarget [12] to identify their possible human mRNA targets. The resulting target genes were then fed to g:Profiler [13]. For evaluating human Gene Ontology (GO) term enrichment, we focused on biological process and molecular function categories potentially affected by the presence of the putative pmiRNAs in the consumers. Both tools were used with default options, except for “Term size” in g:Profiler, which was set between 50 and 500.

***Consensus sequence calculation***

To extract a *consensus* region from the selected sets of sequences, multiple alignment was performed by MUSCLE [14], through a Biopython script [15]. *Consensus* sequences were calculated using the *gap_consensus()* method, with a threshold of 0.7.

**B) REFERENCE LIST OF THE *MATERIALS AND METHODS* SECTION**

1. Leinonen R, Sugawara H, Shumway M, International Nucleotide Sequence Database Collaboration (2011) The sequence read archive. Nucleic Acids Res 39:D19-21. <https://doi.org/10.1093/nar/gkq1019>
2. Stefanizzi FM, Nielsen N, Zhang L, Dankiewicz J, Stammet P, Gilje P, Erlinge D, Hassager C, Wise MP, Kuiper M, Friberg H, Devaux Y, Salgado-Somoza A (2020) Circulating levels of brain-enriched micrornas correlate with neuron specific enolase after cardiac arrest—a substudy of the target temperature management trial. Int J Mol Sci 21:4353. <https://doi.org/10.3390/ijms21124353>
3. Wang X-J, Gao J, Wang Z, Yu Q (2021) Identification of a potentially functional microRNA–mRNA regulatory network in lung adenocarcinoma using a bioinformatics analysis. Frontiers in Cell Dev Biol 9. <https://doi.org/10.3389/fcell.2021.641840>
4. Yang C-K, Hsu H-C, Liu Y-H, Tsai W-S, Ma C-P, Chen Y-T, Tan BC-M, Lai Y-Y, Chang IY-F, Yang C, Yang C-Y, Yu J-S, Liu H (2022) EV-miRome-wide profiling uncovers miR-320c for detecting metastatic colorectal cancer and monitoring the therapeutic response. Cell Oncol 45:621–638. <https://doi.org/10.1007/s13402-022-00688-3>
5. Franco S, Buccione D, Pluvinet R, Mothe B, Ruiz L, Nevot M, Jordan-Paiz A, Ramos L, Aussó S, Morillas RM, Sumoy L, Martinez MA, Tural C (2018) Large-scale screening of circulating microRNAs in individuals with HIV-1 mono-infections reveals specific liver damage signatures. Antivir Res 155:106–114. <https://doi.org/10.1016/j.antiviral.2018.05.008>
6. Andrews S (2010) FastQC: a quality control tool for high throughput sequence data. Babraham Bioinformatics, Babraham Institute, Cambridge, United Kingdom.
7. Langmead B, Trapnell C, Pop M, Salzberg SL (2009) Ultrafast and memory-efficient alignment of short DNA sequences to the human genome. Genome Biol 10:R25. <https://doi.org/10.1186/gb-2009-10-3-r25>
8. Camacho C, Coulouris G, Avagyan V, Ma N, Papadopoulos J, Bealer K, Madden TL (2009) BLAST+: architecture and applications. BMC Bioinform 10:421. <https://doi.org/10.1186/1471-2105-10-421>
9. Pedregosa F, Varoquaux G, Gramfort A, Michel V, Thirion B, Grisel O, Blondel M, Prettenhofer P, Weiss R, Dubourg V, Vanderplas J, Passos A, Cournapeau D, Brucher M, Perrot M, Duchesnay E (2011) Scikit-learn: machine learning in Python. J Mach Learn Res 12:2825–2830.
10. Virtanen P, Gommers R, Oliphant TE, Haberland M, Reddy T, Cournapeau D, Burovski E, Peterson P, Weckesser W, Bright J, van der Walt SJ, Brett M, Wilson J, Millman KJ, Mayorov N, Nelson ARJ, Jones E, Kern R, Larson E, … Vázquez-Baeza Y (2020) SciPy 1.0: fundamental algorithms for scientific computing in Python. Nat Methods 17:261–272. <https://doi.org/10.1038/s41592-019-0686-2>
11. Ward JrJH (1963) Hierarchical grouping to optimize an objective function. J Am Stat Assoc 58:236–244. <https://www.jstor.org/stable/2282967>
12. Dai X, Zhuang Z, Zhao PX (2018) psRNATarget: a plant small RNA target analysis server (2017 release). Nucleic Acids Res 46:W49–W54. <https://doi.org/10.1093/nar/gky316>
13. Raudvere U, Kolberg L, Kuzmin I, Arak T, Adler P, Peterson H, Vilo J (2019) g:Profiler: a web server for functional enrichment analysis and conversions of gene lists (2019 update). Nucleic Acids Res 47:W191–W198. <https://doi.org/10.1093/nar/gkz369>
14. Edgar RC (2004) MUSCLE: multiple sequence alignment with high accuracy and high throughput. Nucleic Acids Res 32:1792–1797. <https://doi.org/10.1093/nar/gkh340>
15. Cock PJA, Antao T, Chang JT, Chapman BA, Cox CJ, Dalke A, Friedberg I, Hamelryck T, Kauff F, Wilczynski B, et al. (2009) Biopython: freely available Python tools for computational molecular biology and bioinformatics. Bioinformatics 25:1422–1423. <https://doi.org/10.1093/bioinformatics/btp163>

**C) CONTINUATION OF THE REFERENCE LIST RELATIVE TO THE MAIN TEXT**

1. Maleki SJ, Crespo JF, Cabanillas B (2019) Anti-inflammatory effects of flavonoids. Food Chem 299:125124. <https://doi.org/10.1016/j.foodchem.2019.125124>
2. Arvey A, Larsson E, Sander C, Leslie CS, Marks DS (2010) Target mRNA abundance dilutes microRNA and siRNA activity. Mol Syst Biol 6:363. <https://doi.org/10.1038/msb.2010.24>
3. McCarroll J, Kavallaris M (2012) Nanoparticle delivery of siRNA as a novel therapeutic for human disease. Nucleus 7:m7G.
4. O’Brien J, Hayder H, Zayed Y, Peng C (2018) Overview of MicroRNA biogenesis, mechanisms of actions, and circulation. Front Endocrinol 9:402. <https://doi.org/10.3389/fendo.2018.00402>
